# Socket‐Shield Technique and Concentrated Growth Factors for Implant Placement in the Esthetic Zone: A Two‐Year Case Report

**DOI:** 10.1002/ccr3.72126

**Published:** 2026-02-26

**Authors:** Shiwei Che, Noor Huda Ismail, Melissa Wan Yun Ooi, Raja Azman Awang

**Affiliations:** ^1^ Prosthodontics Unit, School of Dental Sciences, Health Campus Universiti Sains Malaysia Kubang Kerian Kelantan Malaysia; ^2^ Dr. Che Dental Clinic Luzhou City China; ^3^ Periodontics Unit, School of Dental Sciences, Health Campus Universiti Sains Malaysia Kubang Kerian Kelantan Malaysia

**Keywords:** dental, dental implants, esthetics, growth factors, tooth fracture, wound healing

## Abstract

This clinical report presents a two‐year follow‐up of a case involving a traumatic tooth fracture, successfully managed using a combination of the socket‐shield technique and concentrated growth factors (CGF). The patient, a 26‐year‐old female, experienced fractures in teeth #21 and #22 following a road traffic accident. For the restoration of tooth #22, the socket‐shield technique was employed to preserve the buccal root fragment, maintaining the natural contour of the surrounding soft and hard tissues. The socket was then filled with CGF to promote wound healing and facilitate tissue regeneration. Immediate implant placement was performed without raising a flap, and tooth #21 underwent endodontic treatment followed by full‐coverage crown placement. During the two‐year follow‐up period, clinical and radiographic evaluations were conducted to assess implant stability and tissue preservation. The results indicated favorable outcomes, with minimal bone resorption and stable buccal soft tissue contours. CBCT imaging revealed no significant changes in the alveolar ridge height or thickness. Furthermore, the application of CGF appeared to enhance soft tissue healing, contributing to a stable esthetic outcome in the anterior maxillary region. This case illustrates the use of the socket‐shield technique in combination with concentrated growth factors (CGF) for implant placement in the esthetic zone, where maintenance of peri‐implant soft‐tissue contours is a key objective. In this case, CGF alone was used to manage and seal the peri‐implant gap, without adjunctive particulate grafting, and stable peri‐implant tissues were observed during follow‐up. However, given the technique‐sensitive nature of socket‐shield therapy and the limitations of a single‐case report, further controlled studies are required to clarify long‐term outcomes and reproducibility. Data Access Statement: All relevant data are within the paper and its Supporting Information files.

## Key Clinical Message

Socket‐shield–assisted immediate implant placement, combined with concentrated growth factors for socket sealing, may help maintain peri‐implant soft‐tissue contour in the esthetic zone. In this 2‐year case, the peri‐implant gap was managed with concentrated growth factors alone, achieving stable peri‐implant tissues with meticulous technique.

## 1 Introduction

When the remaining root structure cannot be preserved following dental trauma in the anterior maxillary region, extraction often raises concerns regarding the conservation of the labial bone wall and soft tissues [1]. Following tooth extraction, significant changes occur in the alveolar ridge, including horizontal bone loss of 29%–63% and vertical bone loss of 11%–22% within 6 months. This is partly due to the reduction in blood supply to the post‐extraction area, as the labial bone primarily receives blood from the periodontium. Diminished vascularization affects the bone's ability to maintain its density and volume, leading to resorption. Other factors influencing these changes include the body's natural healing process, which involves bone remodeling, and the loss of the tooth's physical presence, which plays a critical role in stimulating and maintaining alveolar bone [2]. To reduce post‐extractive bone resorption, biomaterials such as collagen sponges support periosteal mechanical integrity, whereas strategies such as immediate restoration with customized healing abutments promote swift epithelialization, thus mitigating myofibroblast activity crucial in early healing stages [3, 4]. Myofibroblast inhibition is particularly important, as these cells contribute to wound contraction and can negatively impact tissue architecture if not controlled during the healing process. Various ridge‐preservation strategies have been proposed to limit post‐extraction remodeling, including socket sealing with collagen‐based materials, placement of xenografts or allografts, and autologous platelet concentrates. In addition, immediate implant placement combined with customized healing abutments or immediate provisionalization has been advocated to support early soft‐tissue stability. Nevertheless, these approaches may not fully prevent facial bone resorption, particularly in thin‐wall sockets, prompting interest in partial extraction therapies such as the socket‐shield technique.

Building on the idea of tissue preservation for esthetic dental implants, the socket shield method, which maintains part of the tooth root, aims to protect nearby bone and soft tissues [5]. The socket‐shield technique, first proposed by Hürzeler et al. [6] aims to preserve rather than compensate for tissue loss and has been effective for implant placement in the esthetic zone. However, this technique is more challenging, technically sensitive, and time‐consuming than the conventional immediate implant placement [7].

The use of Concentrated Growth Factors (CGF) as adjunctive therapy has gained attention for its potential to enhance bone and soft tissue regeneration [8]. The choice between bone grafting and CGF for socket filling remains debatable. This report highlights the specialized protocol, intricacies, and critical considerations that leverage the socket‐shield technique augmented with CGF to manage traumatic tooth fractures in the anterior maxillary region.

## 2 Case Examination and Presentation

**FIGURE 1:**
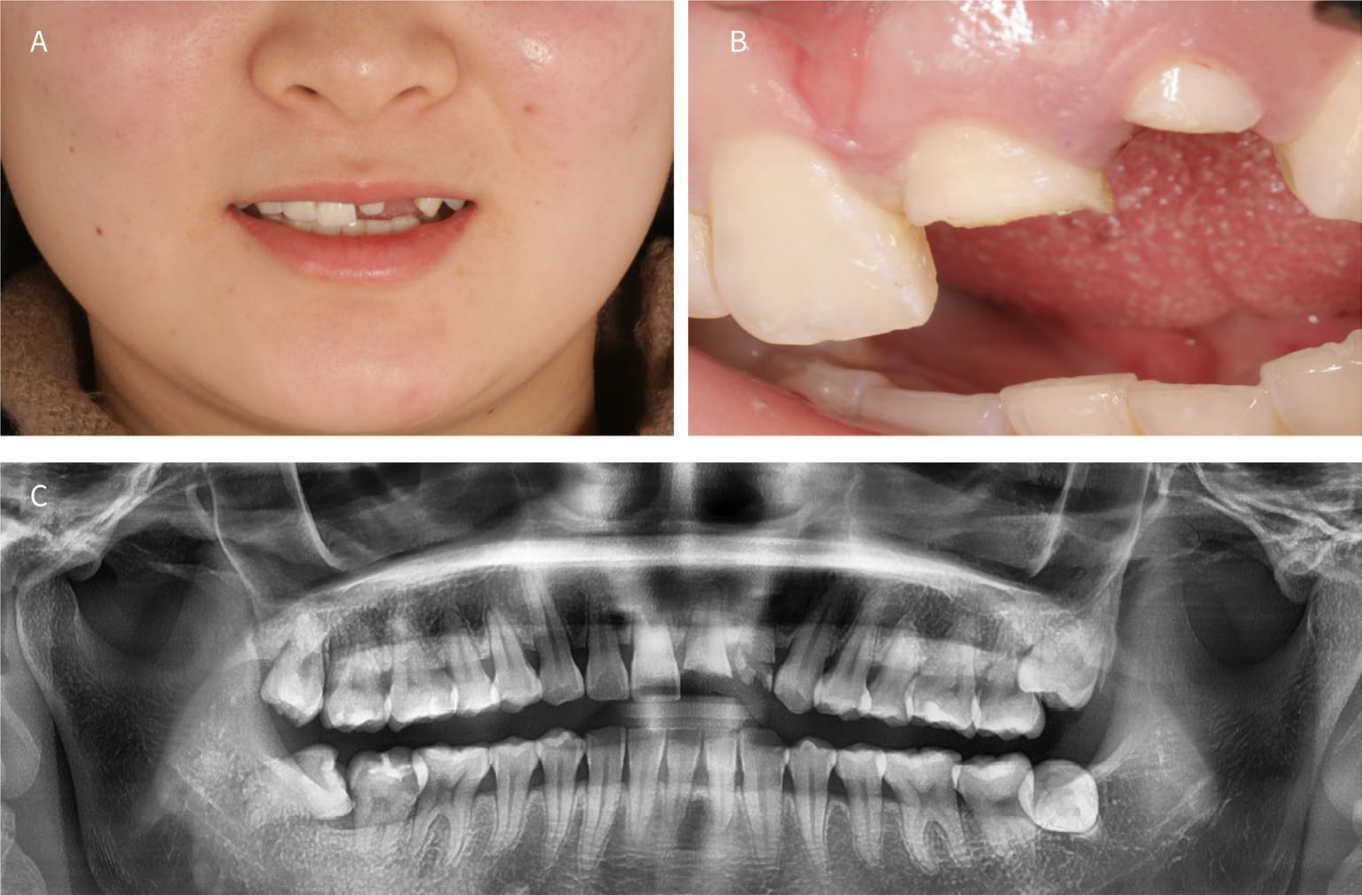
(A) Preoperative smile view; (B) Clinical intraoral examination before surgery; (C) Preoperative panoramic radiograph.

**FIGURE 2:**
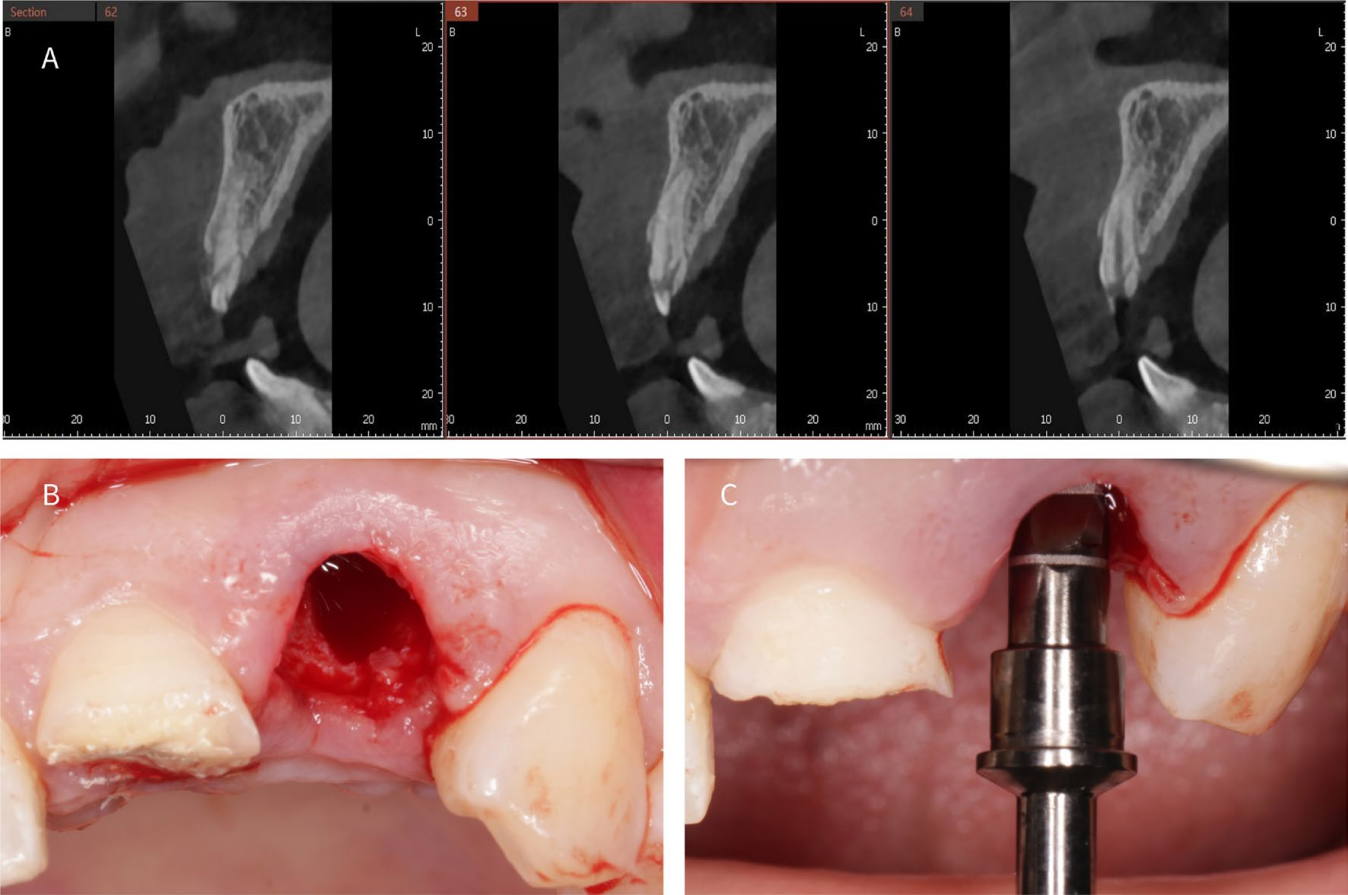
(A) Preoperative CBCT showing an intact but thin facial bone wall at tooth #22. (B) Socket‐shield preparation with the buccal root fragment retained and refined. (C) Sequential implant site preparation using the manufacturer's drilling protocol, with the final drill positioned toward the palatal aspect of the socket.

A 26‐year‐old Chinese female presented with crown fractures of teeth 21 and 22 due to a recent road traffic accident (Figure 1). Clinical examination revealed that tooth #21 retained only one‐third of its crown with an exposed pulp. Panoramic radiographic evaluation revealed that tooth #22 was fractured below the alveolar bone level (Figure 1C). A facial profile photograph indicated a low smile line (Figure 1A). Preoperative 3D cone beam computed tomography (CBCT) further confirmed the preservation of a significant portion of the root of tooth #22 with an intact but thin buccal bone wall (Figure 2A). The patient had no history of smoking or systemic disease. Due to the esthetic factors and the rapid resorption of the buccal bone plate following the extraction of tooth #22, combined with the patient's thin gingival biotype, there were concerns about potential challenges in maintaining the soft tissue architecture. A flapless approach was therefore preferred to minimize disruption of the periosteal blood supply to the facial bone wall [1].

## 3 Methods (Treatment Planning)

After considering various treatment options and the fact that the teeth were traumatized with relatively healthy periodontal tissues, a flapless approach using the socket‐shield technique was chosen for immediate implant placement in tooth #22 [5]. The extraction socket was filled with CGF to promote healing and maintain alveolar ridge volume [9]. Concurrently, tooth 21 underwent endodontic treatment followed by a full‐coverage crown.

Following local anesthesia, the residual crown and root of tooth #22 were carefully removed using a high‐speed handpiece, retaining only the buccal portion of the upper two‐thirds of the root. Root separation was performed using a high‐speed handpiece under copious saline irrigation to minimize heat generation [9]. To ensure ideal conditions for implant placement, detailed attention was directed toward the specific anatomical features and dimensions of the treatment area. The palatal bone wall was preserved with a minimum thickness of 1 mm, and a buccal gap between the implant and the preserved buccal root fragment was maintained to avoid contact and allow socket filling. To prepare the labial shield, the remaining root fragment of tooth #22 was meticulously ground using a high‐speed turbine handpiece. This process involved gradual controlled grinding to preserve the fragment to a thickness of approximately 1.5 mm. The thickness and height of the root fragments were carefully measured using a periodontal probe to ensure that the fragments reached the desired dimensions. It is important to note that (Figure 2B), due to its angle, does not clearly display the thickness of the prepared root fragment. This precise control in grinding is crucial to maintain the stability of the labial shield while ensuring that it is not excessively bulky, which could interfere with implant placement or soft tissue adaptation [5]. The implant surgical kit (Dentium) was used after socket‐shield preparation. Precise location points were established according to the kit's guidelines, and step‐by‐step drilling was performed. The final drill (XFD34 29) was positioned against the palatal wall of the socket to avoid contact with buccal tissues, resulting in a drill depth of approximately 11 mm (Figure 2C).

**FIGURE 3:**
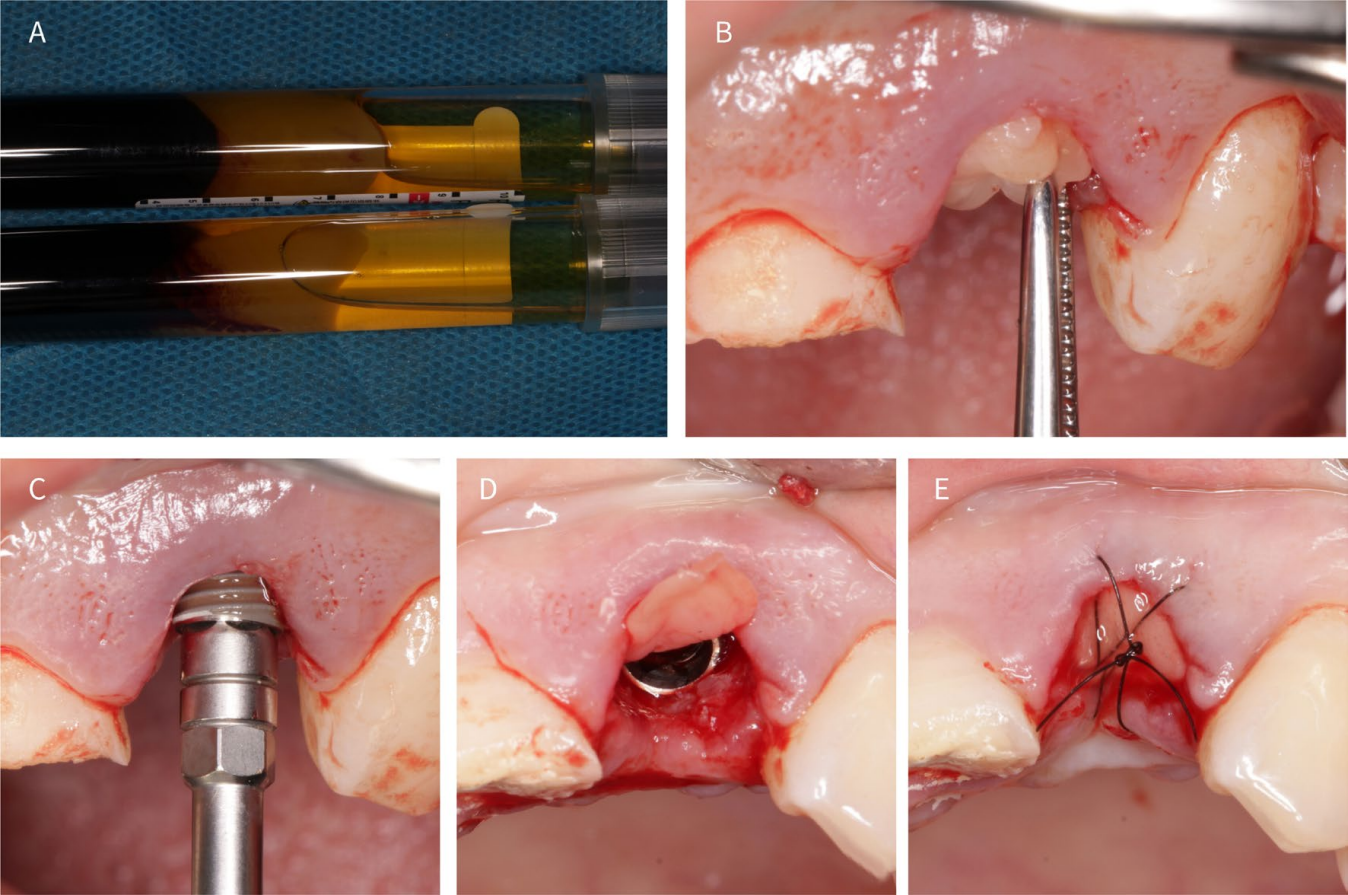
(A) Solid CGF obtained post‐centrifugation; (B) Placing CGF into the socket; (C) Implant placement (Dentium SuperLine FX 36 10 SW), into the prepared site; (D) Association between implant and socket shield. (E) Socket sealing with CGF and suturing.

Two 9 mL samples of autologous blood were drawn from the patient's vein using an anticoagulant‐free vacuum tube. The blood was then subjected to centrifugation using a specialized dental centrifuge (Xiang Qi Dental Centrifuge, China) featuring a pre‐programmed speed setting for the centrifugation process, facilitating the generation of CGF from the blood (Figure 3A). The solid CGF layer was meticulously separated using sterile surgical scissors and positioned in the prepared socket (Figure 3B) [10]. An implant (Dentium; SuperLine FX 36 10 SW) was subsequently placed to achieve a final insertion torque of approximately 25 N. cm (Figure 3C). After implantation, the extraction site was covered with CGF and sutured to promote healing (Figure 3D,E).

**FIGURE 4:**
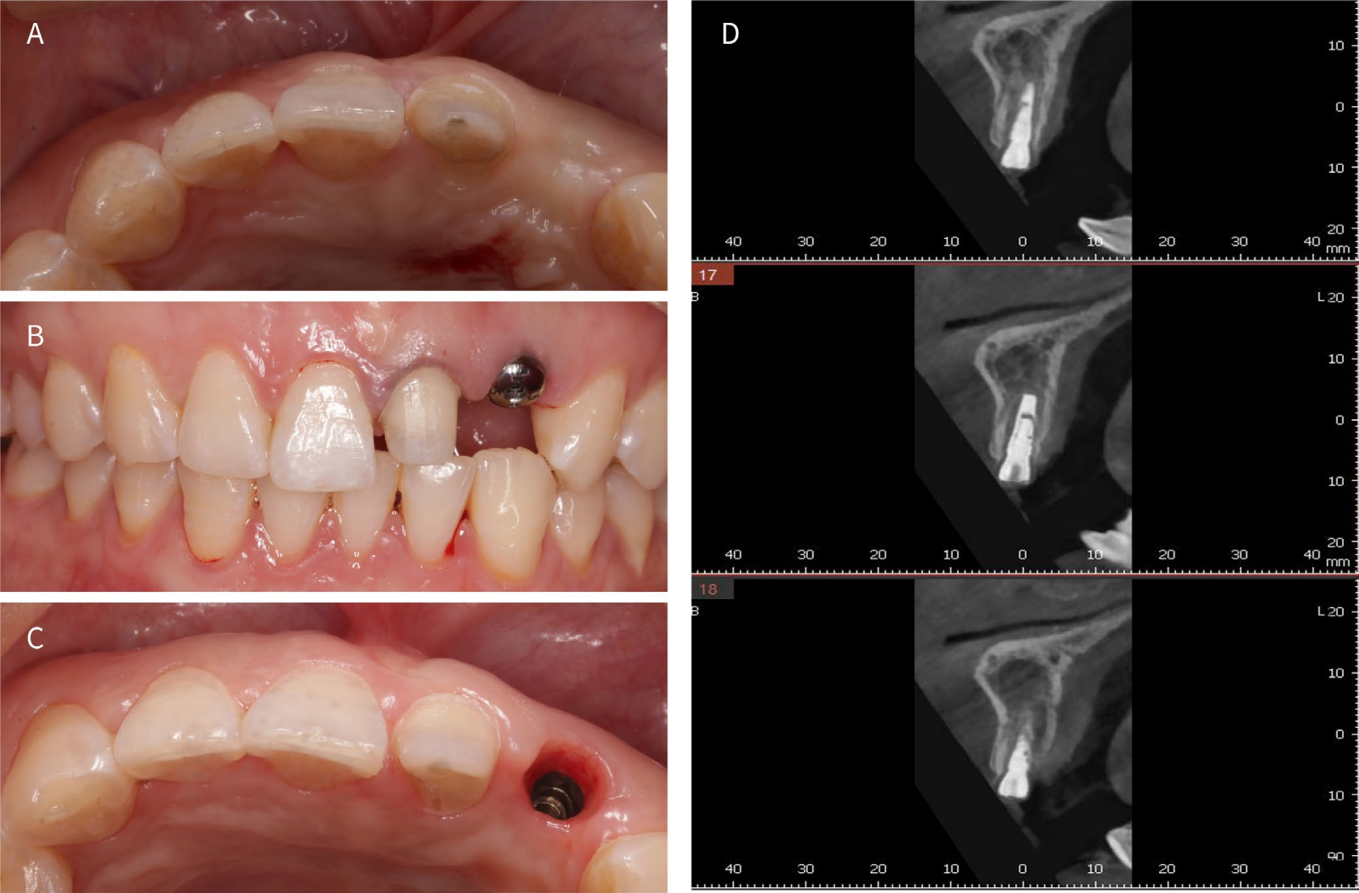
(A) Three‐month follow‐up healing assessment; (B) 1 month after healing abutment placement; (C) Soft‐tissue emergence profile after 1 month of healing abutment placement; (D) No hard‐tissue resorption was detected from the cone beam computed tomography image.

3 months postoperatively, no significant recession was observed in the soft or hard tissues surrounding the surgical site, maintaining a stable buccal contour. After confirming the asymptomatic status of tooth #21, tooth preparation was performed (Figure 4A). 1 month after placing the healing abutment (Dentium; HAB 45 20 35 L), an ideal emergence profile was achieved with minimal recession of the mesial and distal papillae (Figure 4B,C). CBCT imaging revealed that the retained root fragment, positioned buccally to the implant, effectively preserved the buccal bone plate and showed no significant resorption. Additionally, there was no noticeable reduction in the alveolar bone height (Figure 4D).

## 4 Conclusion and Results (Outcome and Follow up)

**FIGURE 5:**
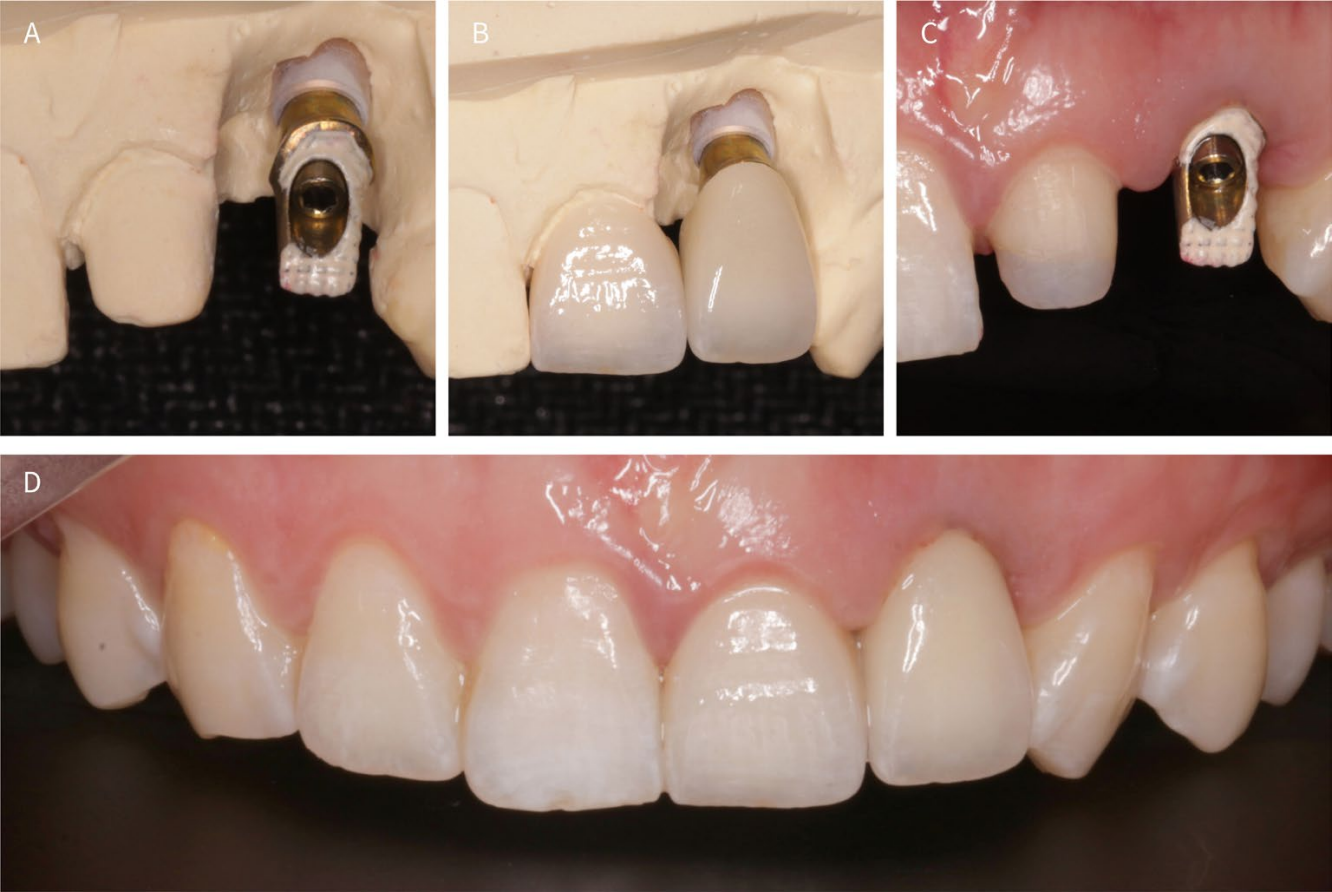
(A) Restorative abutment; (B) Full‐ceramic single crown; (C) Restorative abutment placement; (D) Post‐restoration intraoral views.

**FIGURE 6:**
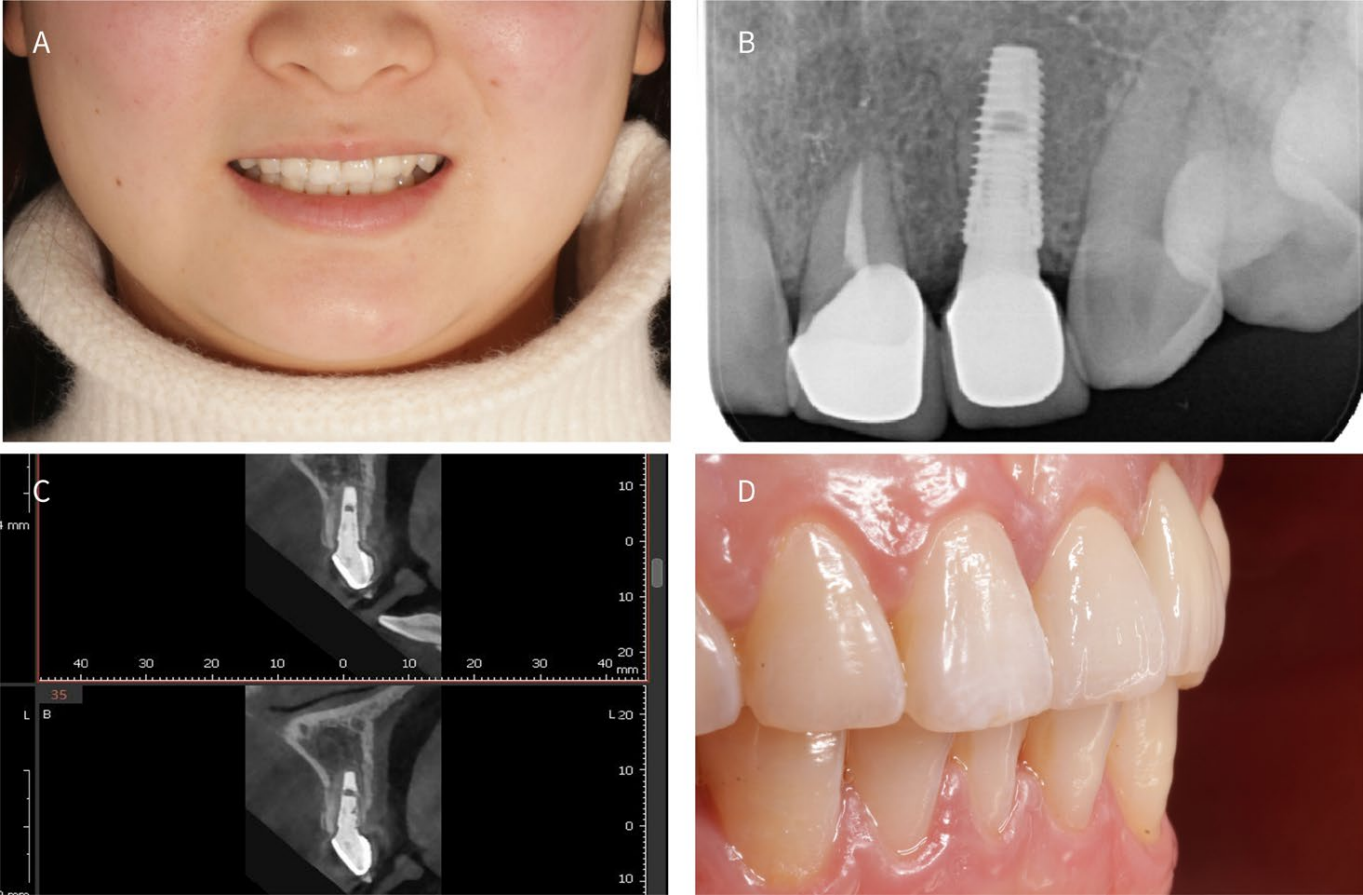
(A) Frontal smile view after definitive restoration placement; (B, C) Post‐restoration radiographic images, no hard‐tissue resorption was detected; (D) Final restoration intraoral views.

After a four‐month postoperative healing period, impressions were taken of teeth #21 and #22 to fabricate ceramic crowns. For tooth #22, a prefabricated abutment (Dentium; DAB 45 25 HL) was customized through milling and subsequently shade‐masked using a composite resin to ensure optimal esthetics (Figure 5A–C). The final positioning of the peri‐implant soft tissues resulted in a relatively satisfactory esthetic appearance, as evaluated clinically and using the Pink Esthetic Score (PES) (Figures 5D and 6A,D) [11]. According to the PES assessment, tooth #22 exhibited incomplete mesial and distal papillae compared with contralateral tooth #12, with only slight differences noted in soft‐tissue color and the level of the soft‐tissue margin [10]. Postoperative radiographs after crown placement revealed that the implant had good osseointegration (Figure 6B,C).

**FIGURE 7:**
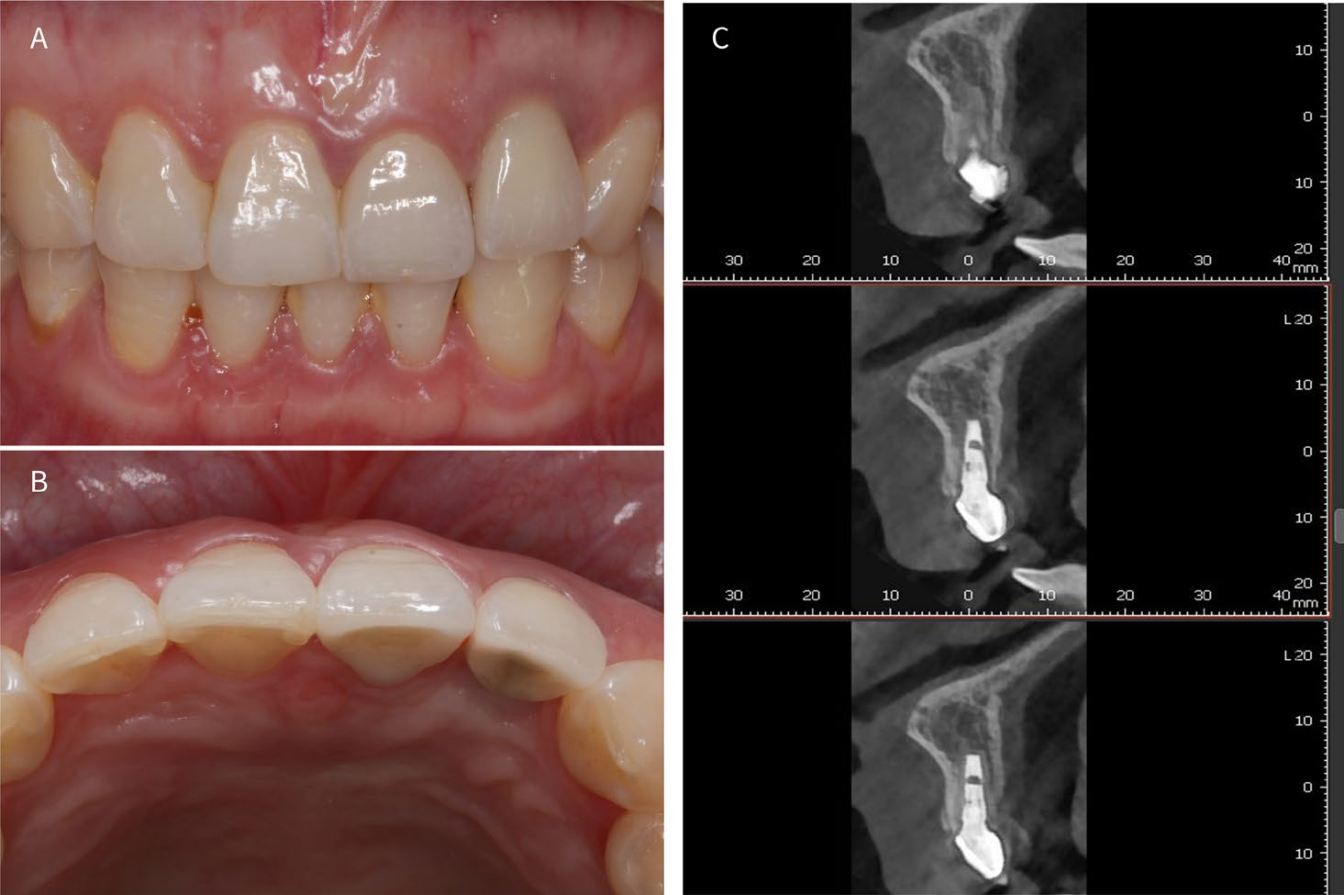
(A, B) Two‐year post‐restoration follow‐up intraoral views; (C) Two‐year follow‐up cone beam computed tomography (CBCT) revealed no significant hard‐tissue resorption observed.

**FIGURE 8:**
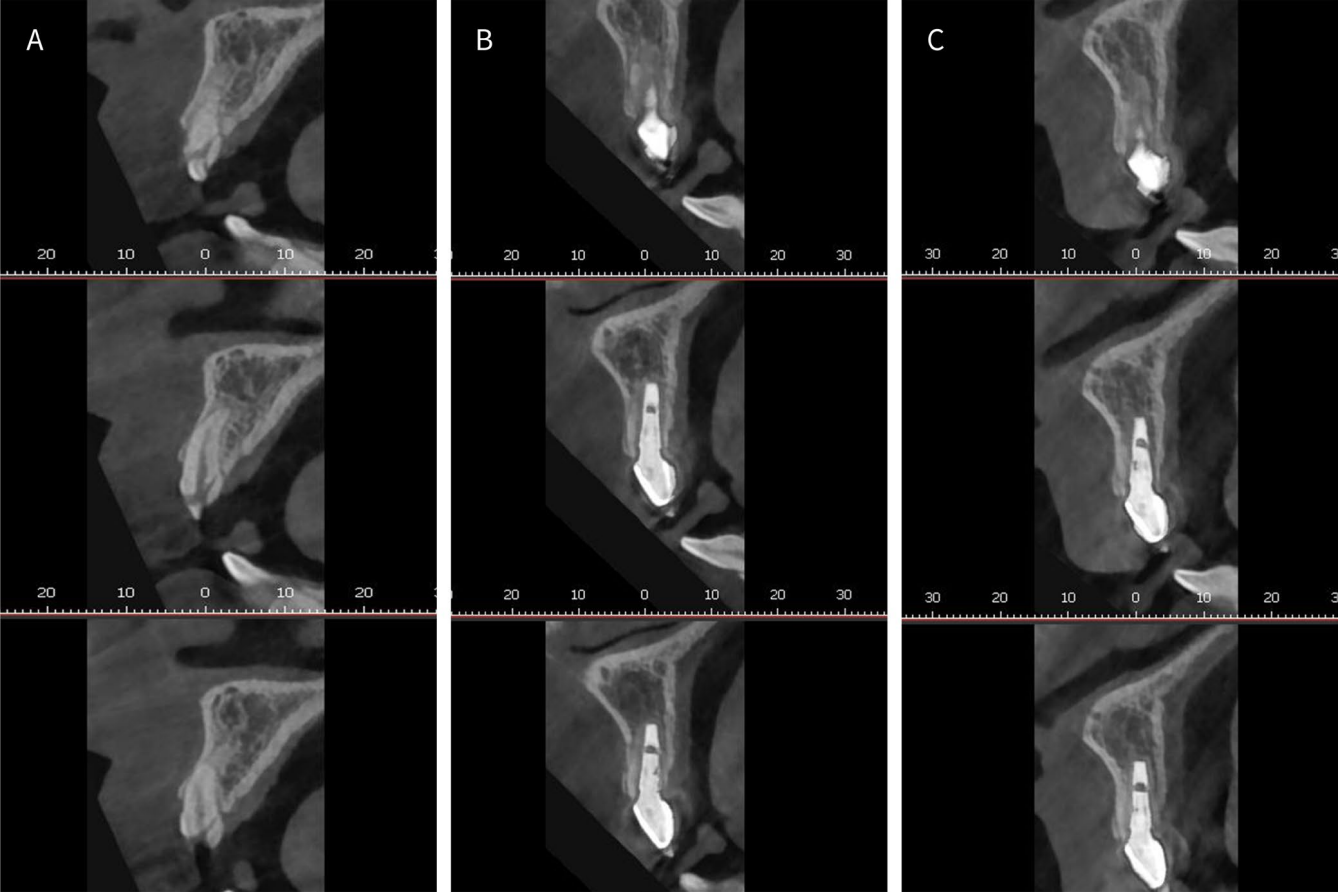
(A) Preoperative CBCT scan; (B) Final restoration CBCT scan; (C) Two‐year follow‐up CBCT scan.

During the two‐year follow‐up, both soft and hard tissues appeared stable (Figure 7A,B). Although slight gingival inflammation was observed, it was likely associated with the patient's oral hygiene practices. Oral hygiene reinforcement and professional prophylaxis were provided during follow‐up to manage gingival inflammation. Nevertheless, even in the presence of gingival inflammation, CBCT images clearly showed a well‐preserved buccal bone wall (Figure 7C). To quantify hard‐tissue stability, linear measurements were performed on CBCT scans at baseline, after definitive restoration, and at the 2‐year follow‐up. Buccal bone plate thickness and ridge width were assessed at standardized levels (1, 3, and 5 mm apical to the crestal reference) using the same cross‐sectional planes and reference points across time points. The measurements indicated limited dimensional changes (all changes < 0.5 mm across time points), consistent with the clinical findings (Figure 8A–C).

## 5 Discussion

The combination of the socket‐shield technique and CGF demonstrated favorable outcomes in this case. During the early stages of wound healing, the application of CGF appears to promote a conducive environment for implant placement. Additionally, preservation of the root shield over the two‐year follow‐up period contributed to the maintenance of the buccal contour, which is crucial in the esthetic zone for dental implant placement [12]. This observation highlights the potential benefits of the socket‐shield technique and its importance in anterior maxillary implantology [13].

The gap between the implant and retained root was effectively managed using CGF, which not only covered the surgical site but also contributed to favorable stability. While some studies have suggested the use of various bone grafts such as allografts, xenografts, and autografts to address such gaps, our exclusive use of CGF in this case yielded satisfactory results [14]. This success can be attributed to CGF's rich growth factor content of CGF, which is known for its ability to accelerate wound healing, particularly in soft tissue, and enhance bone regeneration compared to conventional bone grafting materials.

However, it is essential to recognize that immediate implant placement in the esthetic zone primarily focuses on preserving the tissue contours. The remodeling process over the past three or 6 months may not significantly impact the long‐term results. In this case, the use of CGF alone to fill the gap serves as a viable alternative in similar situations [14]. It is crucial to emphasize that in this single case with a two‐year follow‐up, preservation of the root shield played a pivotal role in maintaining the buccal soft tissues. Compared with traditional techniques that may involve flap elevation for bone augmentation or non‐flap immediate implantation, the socket‐shield technique offers potential advantages in terms of minimally invasive procedures and cost‐effectiveness [15].

Although the socket‐shield technique has demonstrated certain advantages in this immediate implantation case, it is important to acknowledge that the long‐term stability of this approach needs to be substantiated through additional clinical case studies.

The role of concentrated growth factors in bone and soft‐tissue healing has been increasingly discussed. CGF provides a fibrin scaffold enriched with growth factors and cellular components, which may support angiogenesis and tissue maturation and potentially enhance regenerative outcomes in implant‐related procedures. In the context of socket‐shield therapy, management of the gap between the implant and the retained root fragment remains debated. Some authors advocate the use of xenografts or other grafting materials to stabilize the clot and maintain volume, whereas others report satisfactory outcomes without particulate grafting when the shield is stable and the site is adequately sealed. In the present case, CGF was used as the primary socket filler, and stable peri‐implant tissues were observed during the 2‐year follow‐up; however, the isolated contribution of CGF cannot be determined in a single‐case report.

## Author Contributions

**Shiwei Che:** conceptualization, methodology, resources, writing – original draft. **Noor Huda Ismail:** formal analysis, project administration. **Melissa Wan Yun Ooi:** investigation, visualization. **Raja Azman Awang:** supervision, validation, writing – review and editing.

## Funding

The authors have nothing to report.

## Ethics Statement

Informed consent for clinical management was obtained from the patient. Written informed consent for publication of their details was obtained from the patient and was compliant with the clinic.

## Conflicts of Interest

The authors declare no conflicts of interest.

## Data Availability

The data that support the findings of this study are available on request from the corresponding author. The data are not publicly available due to privacy or ethical restrictions.

## References

[1] D. Buser , V. Chappuis , U. C. Belser , and S. Chen , “Implant Placement Post Extraction in Esthetic Single Tooth Sites: When Immediate, When Early, When Late?,” Periodontology 2000 73 (2017): 84–102. 28000278 10.1111/prd.12170

[2] W. L. Tan , T. T. L. Wong , M. C. M. Wong , and N. P. Lang , “A Systematic Review of Post‐Extractional Alveolar Hard and Soft Tissue Dimensional Changes in Humans,” Clinical Oral Implants Research 23 (2012): 1–21, 10.1111/j.1600-0501.2011.02375.x. 22211303

[3] G. B. Menchini‐Fabris , P. Toti , R. Crespi , G. Crespi , S. Cosola , and U. Covani , “A Retrospective Digital Analysis of Contour Changing After Tooth Extraction With or Without Using Less Traumatic Surgical Procedures,” Journal of Clinical Medicine 11, no. 4 (2022): 922. 35207192 10.3390/jcm11040922 PMC8875248

[4] G.‐B. Menchini‐Fabris , S. Cosola , P. Toti , M. Hwan Hwang , R. Crespi , and U. Covani , “Immediate Implant and Customized Healing Abutment for a Periodontally Compromised Socket: 1‐Year Follow‐Up Retrospective Evaluation,” Journal of Clinical Medicine 12 (2023): 2783, 10.3390/jcm12082783. 37109120 PMC10144425

[5] M. Natale , C. M. Soardi , M. H. A. Saleh , et al., “Immediate Implant Placement Using the Socket Shield Technique. Clinical, Radiographic and Volumetric Results Using 3D Digital Techniques. A Case Series,” International Journal of Periodontics & Restorative Dentistry 44, no. 2 (2023): 187–195, 10.11607/prd.6531. 37939278

[6] M. B. Hürzeler , O. Zuhr , P. Schupbach , S. F. Rebele , N. Emmanouilidis , and S. Fickl , “The Socket‐Shield Technique: A Proof‐Of‐Principle Report,” Journal of Clinical Periodontology 37 (2010): 855–862. 20712701 10.1111/j.1600-051X.2010.01595.x

[7] A. Abd‐Elrahman , M. Shaheen , N. Askar , and M. Atef , “Socket Shield Technique vs Conventional Immediate Implant Placement With Immediate Temporization. Randomized Clinical Trial,” Clinical Implant Dentistry and Related Research 22 (2020): 602–611. 32757311 10.1111/cid.12938

[8] F. Tabatabaei , Z. Aghamohammadi , and L. Tayebi , “In Vitro and In Vivo Effects of Concentrated Growth Factor on Cells and Tissues,” Journal of Biomedical Materials Research. Part A 108 (2020): 1338–1350. 32090458 10.1002/jbm.a.36906

[9] L. F. Rodella , G. Favero , R. Boninsegna , et al., “Growth Factors, CD34 Positive Cells, and Fibrin Network Analysis in Concentrated Growth Factors Fraction,” Microscopy Research and Technique 74 (2011): 772–777. 21780251 10.1002/jemt.20968

[10] A. S. Elsayed , E. H. Abo‐Elfetouh , M. Bilal , and A. F. Hassan , “Comparison Between Immediate Implant Placement in the Aesthetic Zone With and Without Socket Shield Technique,” Egyptian Dental Journal 68 (2022): 235–347.

[11] U. C. Belser , L. Grütter , F. Vailati , M. M. Bornstein , H. P. Weber , and D. Buser , “Outcome Evaluation of Early Placed Maxillary Anterior Single‐Tooth Implants Using Objective Esthetic Criteria: A Cross‐Sectional, Retrospective Study in 45 Patients With a 2‐to 4‐Year Follow‐Up Using Pink and White Esthetic Scores,” Journal of Periodontology 80 (2009): 140–151. 19228100 10.1902/jop.2009.080435

[12] X. Wang , G. Wang , X. Zhao , Y. Feng , H. Liu , and F. Li , “Short‐Term Evaluation of Guided Bone Reconstruction With Titanium Mesh Membranes and CGF Membranes in Immediate Implantation of Anterior Maxillary Tooth,” BioMed Research International 2021 (2021): 4754078. 34869763 10.1155/2021/4754078 PMC8635880

[13] A. S. Gharpure and N. B. Bhatavadekar , “Current Evidence on the Socket‐Shield Technique: A Systematic Review,” Journal of Oral Implantology 43 (2017): 395–403. 28604262 10.1563/aaid-joi-D-17-00118

[14] A. Palermo , L. Giannotti , B. Di Chiara Stanca , et al., “Use of CGF in Oral and Implant Surgery: From Laboratory Evidence to Clinical Evaluation,” International Journal of Molecular Sciences 23 (2022): 15164. 36499489 10.3390/ijms232315164 PMC9736623

[15] L. M. Yang , Z. Z. Liu , S. P. Chen , C. Xie , and B. Wu , “The Study of the Effect of Concentrated Growth Factors (CGF) on the New Bone Regeneration of Immediate Implant,” Advances in Materials Research 1088 (2015): 500–502.

